# Harlequin ichthyosis: A case report and literature review

**DOI:** 10.1002/ccr3.6709

**Published:** 2022-12-05

**Authors:** Abhigan Babu Shrestha, Prince Biswas, Sajina Shrestha, Romana Riyaz, Muhammad Hassnain Nawaz, Shumneva Shrestha, Labiba Hossainy

**Affiliations:** ^1^ M Abdur Rahim Medical College Dinajpur Bangladesh; ^2^ Rajshahi Medical College Rajshahi Bangladesh; ^3^ KIST Medical College Patan Nepal; ^4^ Shadan Institute of Medical Sciences and Research Hyderabad India; ^5^ MaharajgunjMedical Campus, Institute of Medicine Tribhuvan University Kathmandu Nepal; ^6^ Department of Pediatrics Shaheed Ziaur Rahman Medical College Hospital Bogra Bangladesh

**Keywords:** ABCA12 mutation, genetic disorder, harlequin ichthyosis, ichthyosis congenital

## Abstract

Harlequin ichthyosis is a rare autosomal recessive disorder occurring in 1: 3,000,000 birth characterized by thick keratin skin with a scaly appearance. Preterm deliveries, early, and consanguinity of marriage are some risk factors. Antenatal checkup of DNA for ABCA12 mutation helps in diagnosis but ultrasonography in places was not available.

## 1 INTRODUCTION

Harlequin ichthyosis (HI) is the most dangerous form of autosomal recessive congenital ichthyosis characterized by the thickening of the keratin part of the baby's skin and a gross thick scaly appearance, which is a triangular or diamond pattern.^1^ The name takes its origin from its characteristic facial appearance as the face is pulled wide open in the manner of a clown's smile. Marked ectropion and eclabium with absent or poorly developed ears and nose, and mobility limitation of joints are some of the clinical features of HI.^1^ As the skin barrier is severally compromised, there is excessive water loss and electrolyte abnormalities followed by temperature dysregulation and increase risk of infections. Because of this reason, HI is usually fatal albeit aggressive management.

## 2 CASE PRESENTATION

A 20‐year‐old pregnant woman was admitted to our pediatrics department for her second pregnancy due to preterm, premature rupture of membrane and obstetric pain. Her gestational age of pregnancy was approximately 36 weeks based on the first day of the last menstrual period. No remarkable complications were seen in the last ultrasound examination (USG) at the 28th week of pregnancy. She had a negative history of consanguinity of marriage, any relevant past medical history and her family members reporting such condition. She denied having any allergy history and was immunized against Tetanus Toxoid and COVID‐19 vaccine. Her first child died due to neonatal jaundice after 5 days of birth via cesarean section a few years ago.

Her physical examinations were all normal and initial conservative treatment was given along with Inj. Dexamethasone 5 mg, 2.5 ampoule IM stat. Then, the patient was counseled for the cesarean section mode of delivery.

**FIGURE 1:**
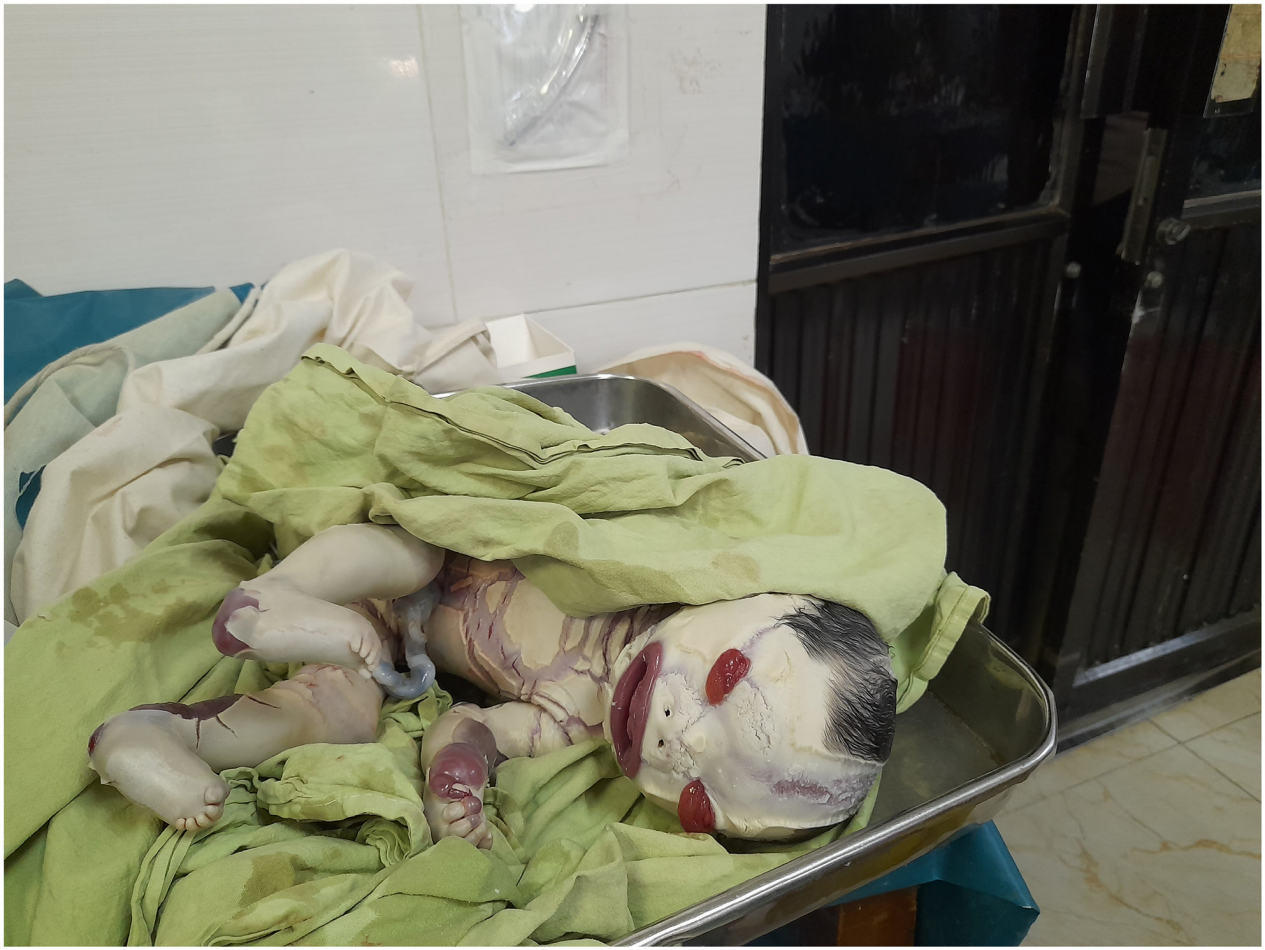
Extensive areas of diamond‐like skin and fissuring characteristic of harlequin ichthyosis.

A female, 2.5 kg, 40 cm height with occipitofrontal circumference of 35 cm baby with Harlequin ichthyosis was born. Figure 1. HI, features were noted by the presence of thick skin with deep fissures, general hyperkeratinization, cyanosis, flat fontanels, ectropion, immature eyes and auricles, bradycardia, bradypnea, and moaning in the physical examination. Her APGAR score was 3 in the 1st minute and the 5th minute. Immediately, she was referred to the neonatal intensive care unit. However, the father of the child was unwilling to the treatment despite several attempts of counseling and had to be discharged with a risk bond.

### 2.1 Patient's parents' perspective

The patient's parents’ belonged to a lower‐middle‐income family and did not have enough money for management. In addition, they mentioned the social stigmata being an important role in not treating the baby. According to their statement, they were not informed about such condition on their previous USG reports nor performed any anomaly scan of the fetus. However, we counseled them about the genetic condition and the need for caution in future pregnancies with the proper test to detect HI.

## 3 DISCUSSION

Harlequin ichthyosis, also called keratosis diffuse fetalis or ichthyosis congenital, is a rare disorder found equally in both sexes^1^ with an overall incidence of 1:300,000 births. Currently, more than 100 cases have been described in the literature.^2^ The first case was reported by Oliver Hart in 1750.^3^ This condition is usually seen in premature babies, early pregnancies, preterm delivery, and more often in consanguineous marriages.^4^, ^5^ In such cases, vaginal delivery is generally the norm while in high‐risk pregnancies, cesarean delivery is performed. In our case, the young mother presented with obstetric pain and preterm premature rupture of the membrane as an obstetric emergency and cesarean delivery was performed. The recurrence of this condition in the subsequent pregnancy is estimated to be 25%.^6^ Hence, it is crucially important to counsel the parents regarding the genetic disorder and its probability of their next conception.

HI is clinically diagnosed at birth with the typical presentation of large, coarse, shiny, yellowish brown, generalized hyper‐keratinized sticky plates resulting in constricted mobility in upper and lower limbs with clasped fists and incurved toes. Later, deep fissures or cracks occur on these hard plates that spread to the dermis. Neonates with HI have growth retardation, eclabium, edema, microcephaly, and ectropion.^7^ Ear appendages, and nostrils look undeveloped and immature.^8^ Additionally, neonates have hypothermia, hypoglycemia, water and electrolytes imbalance, dehydration, infections, sepsis, inadequate feeding habits, renal failure, and more often respiratory complications as a result of limited chest expansion and skeletal abnormalities, eventually leading to death in the early days of life.^9^, ^10^

Later in surviving patients, the hyperkeratotic scales fall off in the first few months, leaving an overlying persistent erythematous skin. In our patient, all these features were present suggestive of HI. Several studies have reported the presence of variation in the ABCA12 (Adenosine‐triphosphate‐Binding Cassette A12) gene encoding a protein for lipid transport in the skin is involved in the pathophysiology of the disease. The ABCA12 gene on chromosome 2 translates a protein involved in keratinocyte lipid transport across the epidermis of the skin which helps in the physiological development of skin and controls the progression of desquamation. The lack of normal ABCA12 function of transportation of lipids from the cytosol to the lamellar granules results in defective skin permeability and accumulation of scales.^11^, ^12^

Prenatal diagnosis is important and helps in the early detection of the disease. Chorionic villous sampling and microscopic analysis of the amniotic fluid cells and USG especially 3D USG for assessment of the shape of the fetal mouth particularly during the early third trimester of pregnancy have been useful for early detection.^13^ Akiyama et al.^14^ specified the first DNA‐based prenatal diagnosis of HI by direct sequential analysis of ABCA12 gene mutation from amniotic fluid cells and established the efficiency of early DNA‐based prenatal diagnosis.

In addition to this, obtaining a detailed account of family history, previous obstetric history, consanguinity between the parents, and the presence of other dermatological disorders in other offspring are equally important.^15^ Postnatal diagnosis includes a skin biopsy that probably shows structural abnormalities of lamellar granules and epidermal keratin expression and is confirmed by testing for ABCA12 gene mutation. Generally, the late phenotypic expression of this condition possesses a challenge and leads to missed or delayed diagnosis on prenatal scans.^12^ Similarly in our case, there was no remarkable complication noted in the last ultrasound examination at 28 weeks of pregnancy. Hence, the gross appearance of the fetus is usually insufficient for diagnosis. To prevent complications and tackle its associated comorbidities, multi‐disciplinary team management is required.

Initial management necessitates monitoring in Neonatal intensive care unit settings (NICU) including supportive therapy to maintain quality of life by use of humidified incubator for monitoring temperature regulation. Intubation helps with the airway and breathing. Maintenance of fluids, electrolytes, and nutritional support through umbilical cannulation as access to peripheral veins becomes difficult. Limb contracture leads to amputation due to the presence of tissue necrosis and gangrene. Hence, it should be a reminder for surgical intervention using autologous skin grafts with the utmost care, physiotherapy, analgesia for painful deep fissures, and proper infection control.^16^ Eye care by artificial tears lubrication and frequent evaluation by ophthalmologists is recommended. Repeated blockage of the ear canal may occur, and debridement is often required. Mild ointments should be applied to make skin soft and soaking with saline compressions helps in desquamation. Further repeated skin culture would be crucial to detect harmful microorganisms.^8^

A comprehensive case series comprising 45 patients assessed by Rajpot et al.^17^ suggested early oral retinoids, aid in the shedding of hyperkeratotic scales, with an overall survival rate of more than 50%. Some studies have shown systemic retinoids can improve survival rates but have both acute and chronic toxicity.^18^ In addition, genetic counseling and molecular investigation of the ABCA12 gene should be considered in subsequent pregnancies as an autosomal recessive disease has been recognized. Studies should further investigate the possible use of immunotherapy.

Critical factors in this patient's management include cooperation and educating the family about the outcomes and options because HI has a social disgrace in our country linked with a lack of knowledge and awareness of the disease and to avoid further endangering the child. Our patient was discharged on request after birth due to non‐compliance by her father.

## 4 CONCLUSION

Harlequin ichthyosis is a severe lethal disorder yet preventable with a proper antenatal checkup. In low‐middle‐income countries like Bangladesh, it is highly important to focus on routine Antenatal follow‐up at least four times. Although DNA analysis for ABC12 mutation will help to diagnose the case in the prenatal period, this might be tough in developing nations. The alternative approach is via USG during the second trimester, but in our case, a single USG could not catch the anomaly hence, repeated USG is highly advisable.

## AUTHOR CONTRIBUTIONS

ABS involved in conceptualization. RR, MHN, and SS involved in writing manuscript. ABS and SS performed manuscript editing and writing. PB collected patient information. LH was the supervisor. All authors reviewed the article and agreed on submission.

## FUNDING INFORMATION

No funding was received for this study.

## CONFLICT OF INTEREST

None declared.

## CONSENT

Informed written consent has been taken from the patient and will be provided on request.
